# The State of Prevention of Atopic Dermatitis: An Updated Review

**DOI:** 10.1111/pde.70157

**Published:** 2026-02-22

**Authors:** Alexandra Kiszluk, Peter Lio

**Affiliations:** ^1^ Department of Medicine University of Tennessee Health Science Center Memphis Tennessee USA; ^2^ Department of Dermatology Northwestern University Feinberg School of Medicine Chicago Illinois USA

**Keywords:** atopic dermatitis, eczema, moisturizer, prevention, probiotic

## Abstract

Atopic dermatitis (AD) is a chronic inflammatory skin disorder that has a high worldwide prevalence and carries substantial psychosocial and financial burden. As such, the prevention of AD is an important area of research. In this review, we provide an update synthesizing relevant clinical trials published from January 2020 through July 2025. Based on the available evidence, moisturizers and probiotics in infancy appear to have the most benefit in the primary prevention of AD compared to other interventions. However, relatively small effects, varying results, and heterogeneity of trial design make firm conclusions impossible at this time.

## 1 Introduction

Atopic dermatitis (AD) is a chronic inflammatory skin disorder marked by pruritic, eczematous patches. Worldwide, an estimated 15%–20% of children are affected by AD [1]. This condition can carry a substantial burden for patients, with one study finding that patients experience an average of nine flares per year [2]. The economic burden is also substantial, with an estimated annual cost of over five billion dollars in the United States [3].

Although the pathogenesis of AD is not entirely understood, it is believed that complex interactions between environmental and genetic factors are involved [4]. The most common genetic associations include loss‐of‐function mutations in filaggrin, a protein responsible for epidermal barrier integrity. Without filaggrin, the resulting disruption of the skin barrier allows penetration of allergens that incite an inflammatory response, contributing to the development of AD. Similarly, certain lipid profiles in the stratum corneum during infancy may contribute to a defective skin barrier, thereby increasing susceptibility to AD [5].

In addition, multiple environmental factors lead to both skin barrier damage and disruption of the microbiome resulting in exposure to bacterial antigens and toxins [6]. In healthy individuals, the skin harbors a more diverse microbiome than in those with AD, while patients with AD have a relative increase in the abundance of *Staphylococcus aureus* . It is hypothesized that toxins from *S. aureus* activate Th2 lymphocytes, mobilize eosinophils, and stimulate IgE production. Changes to the gut microbiome, including decreased abundance of *Bifidobacterium*, are also thought to contribute to the pathogenesis of AD through similar mechanisms [7].

Due to the high prevalence of AD and its impact on quality of life and financial cost, interest in researching strategies for the prevention of AD is growing. In June 2020, Williams and Chalmers published a review of studies seeking to identify effective preventive measures against AD [8]. Here, we provide an update on the state of primary prevention of AD in pediatric patients by summarizing clinical trials published from January 2020 through July 2025. The interventions described aim to maintain the integrity of the skin barrier, promote a balanced microbiome, and prevent inflammation associated with AD.

This review is not a formal scoping or systematic review, but rather a non‐exhaustive narrative review seeking to provide relevant updates since 2020. Although meta‐analyses on the prevention of AD have previously been published, limitations of these papers noted by their respective authors include statistical heterogeneity and low power [9, 10]. Further, they tended to focus on skincare interventions alone, whereas we included studies on all primary preventive measures that have been investigated in trials on AD in the last 5 years.

## 2 Methods

A PubMed search of articles published from January 1, 2020 through July 31, 2025 was completed using the terms ([atopic dermatitis OR eczema] AND prevention). Clinical trials on the primary prevention of AD were included in this review while observational studies, animal studies, laboratory studies, systematic reviews, meta‐analyses, and case reports were excluded.

## 3 Results

A total of 23 trials were located, with ten on moisturizers, six on probiotics, three on dietary supplements, two on dietary habits, three on feeding practices, and one on water filtration. Results are summarized in Table 1.

**TABLE 1:** Summary of results.

Authors/Trial name	Intervention	*N*	Results
Chaoimh et al. – Short‐term topical application to prevent atopic dermatitis (STOP AD) [11]	Moisturizer twice daily for the first 8 weeks of life	321	Incidence of AD after 1 year 32.8% compared to 46.4% among controls (*p* = 0.036)
Simpson et al. – Community‐based assessment of skin care, ezcema, and allergies (CASCADE) [12]	Moisturizer twice daily for the first 2 years of life	1247	Incidence of AD after 2 years 36.1% compared to 43.0% among controls (*p* = 0.02)
Techasatian and Kiatchoosakun [13]	Moisturizer at least once daily for the first 6 months of life	154	60.1% relative risk reduction of AD after 6 months (*p* < 0.001)
Skjerven et al. [14] – Preventing atopic dermatitis and allergies in children (PreventADALL)	Moisturizer at least four times weekly from the second week through the eighth month of life and early introduction of peanut butter, eggs, milk, and wheat	2397	7.4% risk reduction of AD at 3 years of age (95% confidence interval −12.1 to −2.7)
Yuguchi et al. [15]	Moisturizer daily for the first year of life	272	Incidence of AD after 1 year 2.9% compared to 21.2% among controls for infants born during winter months (*p* = 0.0253)
Chalmers et al. – Full barrier enhancement for eczema prevention (BEEP) [16]	Moisturizer daily for the first year of life	1394	No difference in risk of AD compared to control
Harder et al. [17]	Moisturizer at least once daily for the first year of life	100	No difference in risk of AD compared to control
Kottner et al. [18]	Moisturizer once daily for the first year of life	160	No difference in risk of AD compared to control
Ng et al. [19]	Moisturizer twice daily for the first year of life	200	No difference in risk of AD compared to control
Inuzuka et al. [20]	Moisturizer once or twice daily for the first 8 months of life	60	No difference in risk of AD compared to control
Anania et al. [21]	*Bifidobacterium bifidum* PRL2010 daily starting at 36 weeks' gestation	74	No difference in risk of AD compared to control
Komulainen et al. [22]	*Bifidobacterium animalis* ssp. lactis 420 and *Lacticaseibacillus rhamnosus* HN001 (and/or 2.4 g omega‐3 fatty acids) daily starting before 18 weeks' gestation	439	No difference in risk of AD compared to control
Puisto et al. [23]	*Bifidobacterium longum* BL999 with *Lacticaseibacillus paracasei* ST11 or *Lacticaseibacillus rhamnosus* LPR daily starting 2 months prior to expected delivery	46	Incidence of AD at 2 years of age 19.2% compared to 41.3% among controls (*p* = 0.008)
Bellomo et al. [24]	*Bifidobacterium bifidum* PRL2010 daily for the first 6 months of life	413	Incidence of AD at 1 year of age 0% compared to 11.42% among controls (*p* = 0.0130)
He et al. [25]	*Clostridium butyricum* daily for 10 days for the first 2 weeks of life	396	Incidence of AD at 6–36 months of age 8.27% compared to 22.81% among controls (*p* < 0.05)
Robberg et al. [26]	Inactivated *Enterococcus faecalis* and *Escherichia coli* three times daily from 5 weeks to 7 months of age	606	No difference in risk of AD compared to control
Chen et al. [27]	2.4 g omega‐3 fatty acids daily starting at 22–26 weeks' gestation	635	No difference in risk of AD compared to control
El‐Heis et al. – United Kingdom Maternal Vitamin D Osteoporosis Study [28]	1000 IU vitamin D daily starting at 14 weeks' gestation	703	Odds of ad 0.55 (*p* = 0.04)
Del Valle et al. [29]	Extra virgin olive oil and pistachios daily starting before 12 weeks' gestation	1808	Prevalence of AD at 6 years of age 41.3% compared to 69.0% among controls (*p* = 0.005)
Vissers et al. [30]	Complementary feeding starting at 12 weeks (early group) or 17 weeks (late group) of age	255	No difference in risk of AD compared to control
Nicolaou et al. – Allergy Reduction Trial (ART) [31]	Whey‐based partially hydrolyzed formula for the first 6 months of life	331	Incidence of AD after 6 months 11.4% compared to 24.2% among infants fed cow's milk formula (*p* = 0.021)
Di Mauro et al. [32]	Extensively hydrolyzed formula for the first 4 months of life	60	No difference in risk of AD compared to infants fed intact protein formula
Jabbar‐Lopez et al. – Softened Water for Eczema Prevention (SOFTER) [33]	Ion‐exchange water softener installed in home during pregnancy	80	Incidence of AD at 6 months of age 35% compared to 47% among controls (no statistical analysis due to pilot nature of study)

*Note*: Green shading indicates trials demonstrating a statistically significant reduction in atopic dermatitis incidence compared with control; red shading indicates no statistically significant difference.

### 3.1 Moisturization

As skin barrier dysfunction is a central aspect of AD pathogenesis, a number of trials have sought to bolster the skin barrier with topical moisturizers to prevent or delay the onset of AD. In the review by Williams and Chalmers, the authors cited four completed trials and three active trials investigating the role of moisturization in the primary prevention of AD [8]. They concluded that although moisturizers may help prevent AD, sample sizes were small. Since then, we found ten additional completed trials, which included larger cohorts [11, 12, 13, 14, 15, 16, 17, 18, 19, 20].

In the Short‐Term Topical Application to Prevent Atopic Dermatitis (STOP AD) trial, the incidence of AD in infants randomized to application of Aveeno Dermexa Fast & Long Lasting Balm emollient twice daily for the first 8 weeks of life was 32.8% after 1 year, compared to 46.4% in the placebo group (*p* = 0.036) [11]. Similarly, in the Community‐Based Assessment of Skin Care, Ezcema, and Allergies (CASCADE) trial, infants who underwent daily application of one of several popular moisturizers (CeraVe cream, petrolatum, CeraVe Healing ointment, Cetaphil Cream, or Vanicream, in order of frequency used in the trial) had a 36.1% incidence of AD, compared to an incidence of 43.0% among controls, at 2 years of age (*p* = 0.02) [12]. The authors did not provide analysis between the different moisturizer groups.

Additionally, a study in Thailand demonstrated that once‐daily moisturizer application during the first 6 months of life was associated with decreased risk of AD (*p* < 0.001) [13]. There was no difference in risk reduction among the five moisturizers tested (Ezerra lotion, Eucerin Omega Plus Extra Soothing, Eucerin Omega Soothing lotion, Physiogel AI restoring lipid balm, or LyL Hydrating moisturizer).

The Preventing Atopic Dermatitis and Allergies in Children (PreventADALL) trial randomized infants to be bathed with water containing emulsified oil, followed by application of Ceridal cream to the face a minimum of four times weekly from the second week through the eighth month of life [14]. Another group of infants was randomized to a food intervention involving early introduction of peanut butter, eggs, milk, and wheat. A third group received both the skin and food interventions while a fourth group received no intervention. At 3 years of age, infants who underwent both the skin and food interventions experienced a 7.4% decrease in the risk of AD (95% confidence interval −12.1 to −2.7). There was no risk reduction in infants receiving the skin intervention alone.

To investigate the benefits of gentle skin practices, Yuguchi et al. randomized infants to a regimen involving washing the face and body with a washcloth (control group) or by hand (intervention group) [15]. Those washed by hand also underwent daily moisturization. At 1 year of age, the incidence of AD in infants born during winter months was 2.9% in the intervention group and 21.2% among controls (*p* = 0.0253).

Multiple studies found no significant effect of moisturizer application on the rates of AD [16, 17, 18, 19, 20]. Those trials included sample sizes ranging from 60 to 1394 infants and investigated several moisturizers including Doublebase Gel, Diprobase cream, La Roche‐Posay Lipikar Baume AP+ Balsam, HiPP Babysanft Pflegemilch emollient, Fam's Baby moisturizer, and Cetaphil Restoraderm Skin Restoring Moisturizer. There were no differences compared to control after 8–12 months of once‐ or twice‐daily use.

As a whole, moisturization seems extremely promising as a modality to prevent—or at the very least delay—atopic dermatitis. Patient selection, moisturizer selection, and timing may all be important given the heterogeneity of the trial designs, which also precludes meta‐analysis. Though recent trials have built on prior studies by including larger sample sizes, further research should explore the influence of frequency and duration of application, family history, and age on risk reduction.

### 3.2 Probiotics

Dysbiosis of the skin and gut has been associated with AD [6, 7]. The previous review found that probiotic administration during pregnancy may reduce the risk of AD in children by 20% [8]. Since the publication of that review, we identified six trials investigating the role of probiotics in AD prevention [21, 22, 23, 24, 25, 26].

In a study by Anania et al., 39 pregnant women were instructed to take one billion colony‐forming units (CFU) of *Bifidobacterium bifidum* PRL2010 daily starting at 36 weeks' gestation, while 35 women received placebo [21]. There was no significant difference in the prevalence of AD between childern born from the two groups at 3, 6, or 12 months' follow‐up. Likewise, in another trial where pregnant women at less than 18 weeks' gestation received a probiotic capsule containing 10^10^ CFU each of *Bifidobacterium animalis* ssp. lactis 420 and *Lacticaseibacillus rhamnosus* HN001, there was no difference in the odds of AD in children at 1 or 2 years of age compared to placebo [22]. When a separate group of women received both the probiotic capsule and omega‐3 fatty acids, there continued to be no difference in the risk of AD.

Researchers in one study analyzed the gut microbiome of children born to mothers who were administered *Bifidobacterium longum* BL999 with either *Lacticaseibacillus paracasei* ST11 or *Lacticaseibacillus rhamnosus* LPR [23]. Gut microbiota alpha and beta diversity were not significantly different in infants who developed AD by 1 or 6 months of age compared to those who did not, although the prevalence of AD was lower in children whose mothers received probiotics than those who did not (*p* = 0.0008).

Despite that the majority of recent evidence suggests that probiotic administration during pregnancy does not significantly reduce the risk of AD in children [21, 22], research points to a possible benefit of probiotic administration during infancy [24, 25]. In a trial where infants received one billion CFU *Bifidobacterium bifidum* PRL2010 daily for the first 6 months of life, the incidence of AD was significantly lower at 3 (*p* < 0.0001), 6 (*p* = 0.0005), and 12 months (*p* = 0.0130) compared to placebo [24]. Similarly, in another study, the incidence of AD in newborns receiving 15 million CFU *Clostridium butyricum* daily for 10 days during the first 2 weeks of life was significantly lower compared to control at 4–36 months (*p* < 0.05) [25]. However, another study demonstrated that oral administration of an inactivated bacterial lysate comprised of *Enterococcus faecalis* and *Escherichia coli* from 5 weeks to 7 months of age three times daily was not associated with a reduced risk of AD compared to placebo [26]. It is important to note that this formulation does not meet the definition of probiotic, which requires viable bacteria, and therefore would rather be considered a postbiotic. The researchers hypothesized that because the lysate did not contain live cells, colonization could not occur, having no impact on the gut microbiome or the development of AD.

### 3.3 Dietary Supplements

Williams and Chalmers noted in 2020 that fatty acid supplementation during pregnancy has mixed evidence regarding preventive effects on AD [8]. Two trials in the interim have been published investigating this question, both demonstrating no significant benefit [22, 27]. In the first, pregnant women at less than 18 weeks' gestation received 2.4 g omega‐3 long‐chain polyunsaturated fatty acids (LCPUFA) consisting of 9.4% eicosapentaenoic acid (EPA) and 79% docosahexaenoic acid (DHA) until after delivery [22]. At 1 and 2 years of age, there was no difference in the prevalence of AD in children compared to placebo. In the second trial, pregnant women at 22–26 weeks' gestation received 2.4 g omega‐3 LCPUFA consisting of 55% EPA and 37% DHA until after delivery [27]. Compared to placebo, there was no significant difference in prevalence of AD in children through the final follow‐up at 10 years of age.

Based on the results of five clinical trials, Williams and Chalmers also concluded that vitamin D supplementation has no clear benefit in the prevention of AD [8]. One new trial on vitamin D, the United Kingdom Maternal Vitamin D Osteoporosis Study, has been published since [28]. Interestingly, children born to mothers who received 1000 IU vitamin D daily beginning at 14 weeks' gestation demonstrated lower odds of AD at 1 year of age compared to control (*p* = 0.04).

### 3.4 Special Diets

Anti‐inflammatory diets have recently gained popularity in mainstream culture. The review by Williams and Chalmers did not include any studies on the effects of maternal dietary habits [8]. In one new study, the prevalence of AD at 6 years of age in children born to mothers who followed a Mediterranean diet supplemented with extra virgin olive oil (EVOO) and pistachios during pregnancy was 41.3%, compared to 69.0% when mothers followed a Mediterranean diet without supplementation of EVOO or pistachios (*p* = 0.005) [29].

In terms of infants' diet, early exposure to allergenic foods has been hypothesized to decrease risk of atopy. However, the PreventADALL trial found that infants who consumed peanut butter, eggs, milk, and wheat starting at 12 weeks of age did not have a decreased risk of AD compared to controls [14].

### 3.5 Feeding Practices

Based on the available evidence in 2020, Williams and Chalmers concluded that exclusive breastfeeding has no impact on the development of AD [8]. To our knowledge, no new trials have examined the preventive effects of breastfeeding on AD since, although one study in the Netherlands sought to assess the benefits of early complementary feeding in infants born prematurely [30]. Infants born at 30 to 36 weeks' gestation were randomized to begin complementary feeding at a corrected age of 12 (early) or 17 (late) weeks. At 2 years of age, there was no difference in Scoring Atopic Dermatitis (SCORAD) severity between the early and late groups. These findings are consistent with previous studies finding no evidence that the age of initiating complementary feeding is associated with AD [8].

Recently, hydrolyzed formula, rather than formula containing intact protein, has been hypothesized to reduce risk of allergic disease due to smaller particle sizes being less likely to trigger immune responses [34]. The review by Williams and Chalmers did not comment on hydrolyzed formula [8]. In 2022, the Allergy Reduction Trial demonstrated a decreased incidence of AD in full‐term infants fed whey‐based partially hydrolyzed formula compared to those fed cow's milk formula (*p* = 0.021) [31]. Infants with a family history of AD experienced the strongest risk reduction. However, in another study, there was no difference in the incidence of AD between infants fed extensively hydrolyzed formula and those fed intact protein formula [32].

### 3.6 Water Filtration

It has been hypothesized that consumption of and bathing in hard water increase risk of AD due to calcium carbonate disrupting skin barrier integrity [35]. No studies investigating the benefits of soft water were included in the review by Williams and Chalmers [8]. In 2022, researchers in the Softened Water for Eczema Prevention (SOFTER) trial explored this question by installing an ion‐exchange water softener in the homes of 17 pregnant women [33]. 35% of their offspring were diagnosed with AD by 6 months of age, compared to 47% of children whose mothers did not have a water softener. However, no statistical analysis was conducted due to the pilot nature of the study.

## 4 Conclusions

Based on current data, early moisturizer use has the most evidence supporting its potential to prevent AD compared to other interventions [11, 12, 13, 14, 15]. The moisturizers recently investigated in clinical trials may exert their preventive effects by strengthening the skin barrier, and they contain a wide variety of ingredients including ceramides, colloidal oatmeal, petrolatum, glyceryl stearate, glycerin, spent grain wax, and shea butter [11, 12, 13, 14]. Although several recent trials demonstrated moisturization to be beneficial in preventing AD, it is important to note that multiple trials also showed that moisturizers led to no difference in the incidence of AD compared to control [16, 17, 18, 19, 20]. For this reason, to clarify the potential of moisturization to prevent AD, further research must elucidate whether variations in factors including duration of treatment, age at initiation, specific moisturizer formulation and ingredients, and family history affect results. It is also important to note that varying adherence to the treatment regimens among participants may have led to some trials demonstrating benefit while others did not. Further, the follow‐up period tended to be shorter in those studies demonstrating no benefit, highlighting the need for future trials to follow children for longer time periods. Among the interventions appearing to show no benefit in the prevention of AD are early complementary feeding and maternal supplementation of omega‐3 fatty acids [22, 27, 30]. Though no clinical trials to our knowledge have examined the preventive effects of breastfeeding on AD in the last 5 years, prior analyses suggest that breastfeeding does not help prevent AD [8]. The administration of probiotics during pregnancy also appears to have little effect [21, 22], although when given to infants, probiotics have demonstrated promising evidence in reducing risk of AD, possibly due to their ability to support gut microbial diversity [24, 25].

The effect of vitamin D supplementation continues to be a developing area of research. Maternal supplementation of vitamin D may prevent AD in children by decreasing Th2 responses and regulating the skin microbiome [28]. Additional studies are needed to clarify this effect and determine whether administration of vitamin D during infancy may offer increased benefit compared to maternal supplementation.

One investigative focus that was not addressed in any study from our search is whether bathing frequency may affect the development of AD in infancy. Although one recent trial has been published comparing the severity of AD in children and adults who bathe weekly versus daily, the researchers did not examine primary prevention, as participants already had a diagnosis of AD prior to enrollment [36]. Another topic for future study could be the role of vernix in the pathogenesis of AD. Because vernix contains enzymes involved in epidermal development and promotes microbial balance in infant skin [37], determining the optimal timing of removal after birth could help maximize these benefits and potentially reduce the risk of AD.

In conclusion, future research on the interventions described in this review should address the most appropriate time to begin the intervention as well as the duration necessary to prevent AD. It would also be useful to examine whether preventive effects are greater in high‐risk children with a family history of AD and whether combinations of interventions could exert synergistic effects. Particularly for moisturizers and oral probiotics, studies should explore which ingredients and concentrations/doses are most beneficial. Additionally, interventions should be easy for patients to implement. The growing body of knowledge on these evidence‐based approaches represents exciting advancement in this area of research.

## Conflicts of Interest

A.K. reports no relevant disclosures. P.L. reports being on the speaker's bureau for AbbVie, Arcutis, Eli Lilly, Galderma, Hyphens Pharma, Incyte, La Roche‐Posay/L'Oreal, Pfizer, Pierre‐Fabre Dermatologie, Regeneron/Sanofi Genzyme, Verrica; reports consulting/advisory boards for Alphyn Biologics, AbbVie, Almirall, Amyris, Apogee, Arcutis, ASLAN, Astria Therapeutics, Boston Skin Science, Bristol‐Myers Squibb, Burt's Bees, Castle Biosciences, Codex Labs, Concerto Biosci, Dermavant, Eli Lilly, Galderma, Kenvue, LEO Pharma, Lipidor, L'Oreal, Merck, Micreos, MyOR Diagnostics, Pelthos Therapeutics, Phyla, Regeneron/Sanofi Genzyme, Sibel Health, Skinfix, Soteri Skin, Stratum Biosciences, Sun Pharma, Theraplex, Thimble Health, UCB, Unilever, Verdant Scientific, Verrica, Yobee Care. Stock options with Alphyn Labs, Codex Labs, Concerto Biosci, Soteri Skin, Stratum Biosciences, Thimble, Yobee Care, Verdant Scientific. In addition, Dr. P.L. has a patent pending for a Theraplex product with royalties paid and is a Board member and Scientific Advisory Committee Member emeritus of the National Eczema Association.

## Data Availability

Data sharing not applicable to this article as no datasets were generated or analysed during the current study.
